# Minimization of heavy metal toxicity in radish (*Raphanus sativus*) by strigolactone and biochar

**DOI:** 10.1038/s41598-024-64596-2

**Published:** 2024-06-13

**Authors:** Khurram Shahzad, Subhan Danish, Sidra Mubeen, Khadim Dawar, Shah Fahad, Zuhair Hasnain, Mohammad Javed Ansari, Hesham S. Almoallim

**Affiliations:** 1 Department of Soil Science, University College of Dera Murad Jamali, LUAWMS, Dera Murad Jamali, Balochistan Pakistan; 2 Department of Soil Science, Faculty of Agricultural Sciences and Technology, Bahauddin Zakariya University, Multan, Punjab Pakistan; 3 Department of Chemistry, The Women University Multan, Multan, 66000 Pakistan; 4 Department of Soil and Environmental Science, The University of Agriculture Peshawar, Peshawar, Pakistan; 5 Department of Agronomy, Abdul Wali Khan University Mardan, Khyber Pakhtunkhwa, 23200 Pakistan; 6 Department of Natural Sciences, Lebanese American University, Byblos, Lebanon; 7 Department of Agronomy, Pir Mehr Ali Shah Arid Agriculture University, Rawalpindi, Pakistan; 8 Department of Botany, Hindu College Moradabad (MJP Rohilkhand University Bareilly), Moradabad, 244001 India; 9 Department of Oral and Maxillofacial Surgery, College of Dentistry, King Saud University, PO Box-60169, 11545 Riyadh, Saudi Arabia

**Keywords:** Antioxidant activity, Biochar, Chlorophyll content, Cadmium, Growth attributes, Strigolactone, Plant physiology, Plant sciences, Plant stress responses, Abiotic

## Abstract

Due to the high solubility of Cd in water, it is considered a potential toxin which can cause cancer in humans. In plants, it is associated with the development of oxidative stress due to the generation of reactive oxygen species. To overcome this issue, the roles of different plant hormones are vital. Strigolactones, one of such natural plant hormones, show promise in alleviating cadmium toxicity by mitigating its harmful effects. Acidified biochar (AB) can also effectively mitigate cadmium toxicity via ion adsorption and pH buffering. However, the combined effects of strigolactone and AB still need in-depth investigations in the context of existing literature. This study aimed to assess the individual and combined impacts of SLs (0 and 25 µM) and AB (0 and 0.75% w/w) on radish growth under Cd toxicity, i.e., 0 and 20 mg Cd/kg soil. Using a fully randomized design (CRD), each treatment was administered in four replicates. In comparison to the control under 20 mg Cd/kg soil contamination, the results showed that 25 µM strigolactone + 0.75% AB significantly improved the following: radish shoot length (~ 17%), root length (~ 47%), plant fresh weight (~ 28%), plant dry weight (~ 96%), chlorophyll a (~ 43%), chlorophyll b (~ 31%), and total chlorophyll (~ 37%). It was also noted that 0.75% AB was more pronounced in decreasing antioxidant activities than 25 µM strigolactone under 20 mg Cd/ kg soil toxicity. However, performing 25 µM strigolactone + 0.75% AB was far better than the sole application of 25 µM strigolactone and 0.75% AB in decreasing antioxidant activities in radish plants. In conclusion, by regulating antioxidant activities, 25 µM strigolactone + 0.75% AB can increase radish growth in cadmium-contaminated soils.

## Introduction

Industrialization and urbanization have accelerated heavy metal pollution. Among different heavy metals, cadmium (Cd) accumulation seriously threatens the environment and human health^1^. Cadmium accumulates in soil, hindering plant growth. Following its exposure, plants experience detrimental effects on various physiological, morphological, and metabolic traits, ultimately resulting in plant mortality^2,3^. Specifically, Cd toxicity interferes with plant development, inhibits nutrient absorption, and disrupts photosynthesis. Additionally, it triggers an increase in the production of reactive oxygen species (ROS) and modifies antioxidant activities^4^. In addition to above excessive Cd in crops also damages human health and caused diseases, i.e., anemia and lung cancer. To overcome this issue phytohormones can play a vital role^4,5^.

Phytohormones are crucial in orchestrating various aspects of plant life, including growth, development, and responses to stress. They govern fundamental functions such as cell division, shaping of plant structures, and adaptation to the environment^6–8^. Among different phytohormones, strigolactones (SLs), is a recently identified phytohormone, have showcased potential applications in plant growth and development^9^. The positive effect of SLs on food security and plant productivity, particularly in mitigating abiotic or biotic stresses such as high salinity, extreme temperatures, drought, and heavy metals, underscores their role in promoting agricultural sustainability^10,11^. SLs mitigate the phytotoxic effects of cadmium (Cd) on photosynthetic pigments and gas exchange parameters, restoring tomato plant growth^12^. Additionally, supplementation with SLs stimulates the plant defense system by boosting the activity of antioxidant enzymes and enhancing the levels of ascorbate and glutathione^13^.

On the other hand, biochar, produced from biomass pyrolysis, enhances soil health by improving organic matter and water retention while reducing nutrient loss^14–16^. It effectively sorbs heavy metals and boosts soil microbial activity^1,17^. With its high surface area and structured carbon matrix, biochar acts as a potent pollutant sorbent. It contains functional groups i.e., thiol (–SH), carboxyl (–COOH), phosphate (–PO_4_), amino (–NH_2_) and hydroxyl (–OH) that bind metals and facilitates the reduction of toxic metals uptake^18,19^.

*Raphanus sativus*, also known as radish, is a nutritious vegetable rich in essential vitamins, minerals, and dietary fiber, and contains antioxidants like anthocyanins, offering numerous health benefits^20^. Radishes contribute diversity, flavor, and color to culinary creations, making them a favored ingredient in a wide array of dishes. Beyond the kitchen, radishes play a crucial role in agriculture. With their rapid growth cycle and ability to thrive year-round, they serve as an important rotational crop. This not only improves soil health but also disrupts disease cycles. Through crop rotation, radishes assist in controlling weeds and pests, enhancing soil quality, and enriching nutrients for subsequent crops^21^. Radishes are frequently employed as indicator plants in studies investigating the toxicity of heavy metals^22^.

As limited literature is available on combined use of SLs and deashed biochar for their usage for alleviation of Cd stress, currents study aims to explore their individual and combined effects on radish growth. The novelty of current study lies in exploring the influence of SLs and AB on growth and antioxidants activities of radish cultivated in normal and Cd contaminated soils. We hypothesized that the combined application of AB and SLs can improve radish growth, chlorophyll contents while decrease electrolyte leakage and antioxidants activities by minimizing Cd stress in radish.

## Material and methods

### Experimental site and soil characterization

The experiment was conducted at in the research area of ResearchSolution. The soil was collected randomly from the top 15 cm depth of the research area. After sun-drying, it was sieved through a 2 mm sieve and characterized for its physicochemical properties (see Table 1). The irrigation water was collected after 30 min of tap water flow for characterization.

**Table 1:** Pre-experimental soil, biochar, and irrigation characteristics.

Soil	Values	Biochar	Values	Irrigation	Values
pH*s*	8.15	pH	6.57	pH	7.02
EC*e* (dS/m)	4.56	EC*e* (dS/m)	5.73	EC*e* (dS/m)	535
Organic matter (%)	0.60	Fixed carbon (%)	50	Carbonates (meq./L)	0.00
AK (mg/kg)	132	Volatile Matter (%)	15	Bicarbonates (meq./L)	4.86
Extractable phosphorus (mg/kg)	6.41	Ash Content (%)	35	Chloride (meq./L)	0.00
Total nitrogen (%)	0.030	Total nitrogen (%)	0.28	Ca + Mg (meq./L)	2.02
Silt (%)	40	Total potassium (%)	0.75	Sodium (mg/L)	119
Sand (%)	30	Total phosphorus (%)	0.48		
Clay (%)	30	Surface area (m^2^ /g)	400		
Texture	Clay Loam	Cation exchange capacity (meq./100 g)	> 275		

*SLs* Strigolactone GR24, a product with CAS Number 76974-79-3 and Catalog No. CS-0103265, was purchased from ChemScene LLC in Multan.

### Acidified biochar

The leaves waste was collected from local mango orchard. After being sun-dried, the obtained waste was pyrolyzed at 470 ± 8 °C in an aerobic environment. After the pyrolysis process, the material was left to cool down before being crushed and ground to produce particles smaller than 2 mm in size. To make acidified biochar, 60 ml concentrated sulfuric acid was mixed in 1 kg of biochar. Subsequently, the acidified biochar (AB) was again dried in sun and then appropriately stored in plastic jars. The properties of biochar are provided in Table 1.

### Pot filling and sowing

Acidified biochar (0 and 0.75% w/w) was mixed manually in soil, and then the mixture of AB and soil was filled into plastic bags (20 cm width and 45 cm in depth). In every plastic bag 10 kg soil was filled as per treatment plan. Each pot initially had five radish seeds sown, later 2 seedlings were retained by thinning following germination..

### Treatment plan

There were 2 levels of SLs, i.e., control (0 µM) and 20 µM. These levels were applied as a foliar spray when the seedlings become 21 days old. Similarly, biochar was applied as 0 and 0.75% w/w in soil. For development of Cd toxicity potassium dichromate was applied so that 20 mg Cd/kg soil toxicity can be achieved. Before experiment conduction, soil was incubated (25 ± 3 °C) and mixed on a regular basis by maintaining 60% field capacity moisture w/w. However, in normal soils no additional cadmium sulfate was added. It was just incubated (25 ± 3 °C) by maintaining 60% field capacity moisture w/w.

### Harvesting and data collection

After 48 days of sowing, the plant's growth attributes data were measured using an analytical grade balance. Samples were subjected to 70 °C heating for 48 h to determine dry weight in an oven.

### Relative water content

The protocol outlined by^23^ was used to determine the RWC of freshly harvested leaves. Leaf samples were collected, weighed, and placed in petri dishes containing distilled water. The turgid weight was documented, and the final dry weight was determined after oven drying.

$$RWC\,\left( \% \right)\, = \,\left( {FW\, - \,DW} \right){/}\left( {TW\, - \,DW} \right)\, \times \,100$$

### Chlorophyll contents and carotenoids

The study utilized Arnon^24^ procedure to assess chlorophyll (a, b, and total) content in freshly harvested leaves, absorbance was measured at specific wavelengths of 663 nm, 645 nm for chlorophyll, and 470 nm for carotenoids^25^.

### Antioxidants

To evaluate SOD activity, we utilized nitro blue tetrazolium (NBT), and the absorbance was measured at 560 nm to determine the final reading^26^. POD activity was assessed by observing the oxidation of guaiacol, with the absorbance recorded at 470 nm for analysis^27^. CAT activity was assessed by observing the degradation of hydrogen peroxide, which led to a decrease in absorbance at 240 nm for analysis^28^. We noticed the oxidation of ascorbate in the presence of H_2_O_2_, observing a decrease in absorbance at 290 nm^29^. The study measured the level of MDA, a dependable indicator of lipid peroxidation, by reacting the sample extract with thiobarbituric acid, leading to the formation of a colored complex, and then measuring its absorbance at 532 nm. Glutathione reductase activity was evaluated by assessing the rate of NADPH oxidation, with the decrease in absorbance at 340 nm observed for analysis^30^. Glutathione (GSH) was analyzed by adding 5% sulfosalicylic acid to the homogenate, followed by centrifugation for 10 min at 12,000×*g*. The resulting supernatant was then combined with 100 mM phosphate buffer and 5,5′-dithiobis(2-nitrobenzoic acid) before being measured spectrophotometrically at 412 nm^31^. To assess ascorbate (AsA) levels, an equal volume of 10% trichloroacetic acid was added to the homogenate, followed by centrifugation for 10 min at 12,000×*g*. The supernatant obtained was used for the spectrophotometric determination of AsA at 525 nm, following the described method^32^.

### Electrolyte leakage

Electrolyte leakage was performed by dipping 1 cm diameter leaf sections in test tubes containing 20 ml of deionized water. The tubes were incubated at 25 °C for 24 h, then measured for electrical conductivity (EC1). After a 20-min heat treatment in a 120 °C water bath, the 2nd electrical conductivity (EC2) was recorded^33^.

### Free proline

Free proline was quantified using a method outlined by^34^, followed by extraction using sulfosalicylic acid, glacial acetic acid and ninhydrin solutions, heating at 100 °C, and adding 5 ml of toluene. The absorbance of the toluene layer was measured at 520 nm.

### Statistical analysis

The data was subjected to conventional statistical analysis^35^. OriginPro software was used to do a two-way ANOVA. OriginPro was used to carry out paired comparisons, convex hull, and hierarchical cluster analysis^36^.

### Ethics approval and consent to participate

We all declare that manuscript reporting studies do not involve any human participants, human data, or human tissue. So, it is not applicable. We confirmed that all methods were performed in accordance with the relevant guidelines/regulations/legislation.

## Results

### Growth attributes

**Figure 1:**
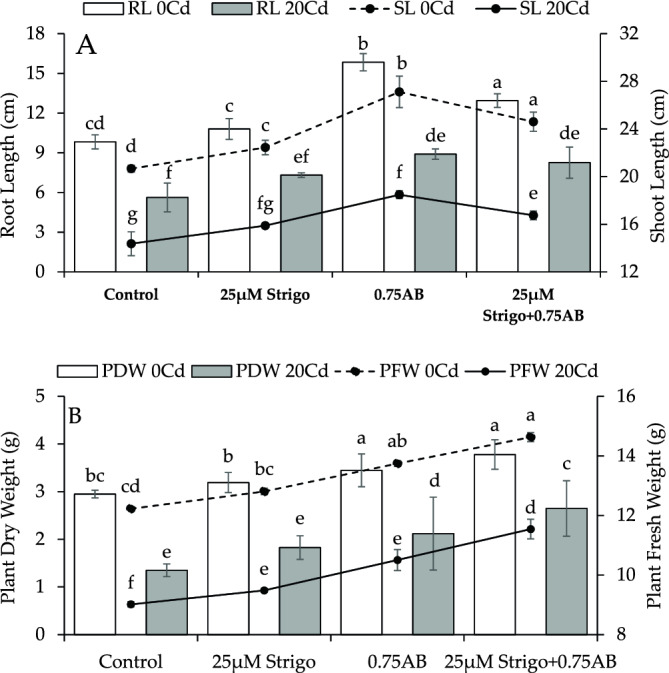
The impact of SLs and biochar on radish shoot and root length (**A**), plant fresh and dry weight (**B**) grown in no Cd and 20Cd stress. The graph shows the average of 4 replicates with±, where significant differences (*p* ≤ 0.05) are indicated by distinct letters on bars, determined by the Tukey test.

The application of 25 µM SLs resulted in ~ 9% and 25 µM SLs + 0.75% AB showed a ~ 19% increase in shoot length over the control under no Cd stress. Compared to control, 0.75% AB exhibited a ~ 31% enhancement in shoot length at no Cd stress. In 20Cd stress, the treatment SLs showed a ~ 11%, 25 µM SLs + 0.75% AB led to ~ 17%, while 0.75% AB resulted in a ~ 29% improvement in shoot length over the control (Fig. 1A).

At no Cd stress, applying 25 µM SLs, 0.75% AB and 25 µM SLs + 0.75% AB led to a ~ 10, ~ 61 and ~ 32% enhancement respectively in root length than control. Under Cd stress (20Cd), an improvement of ~ 30, ~ 58, ~ 47% was observed in root length where 25 µM SLs, 0.75% AB and 25 µM SLs + 0.75% AB were applied as treatment compared to control respectively (Fig. 1A).

An enhancement of ~ 5, ~ 12 and ~ 20% in plant fresh weight was noted in 25 µM SLs, 0.75% AB and 25 µM SLs + 0.75% AB than control respectively. In case of 20Cd stress, SLs resulted in a ~ 5% while 25 µM SLs + 0.75% AB caused ~ 28% improvement in plant fresh weight over control. On the other hand, AB treatment showed ~ 17% improvement in plant fresh weight compared to control under 20Cd stress (Fig. 1B).

In no Cd stress, the addition of 25 µM SLs resulted in ~ 8% improvement in the plant dry weight than control. When 25 µM SLs + 0.75% AB and 0.75% AB were applied, the plant dry weight exhibited a ~ 28 and ~ 17% enhancement over control under no Cd stress. In the presence of 20Cd stress, SLs treatment caused ~ 35%, 25 µM SLs + 0.75% AB ~ 96% and 0.75% AB ~ 57% rise in plant dry weigh over the control (Fig. 1B).

### Chlorophyll and carotenoid contents

**Figure 2:**
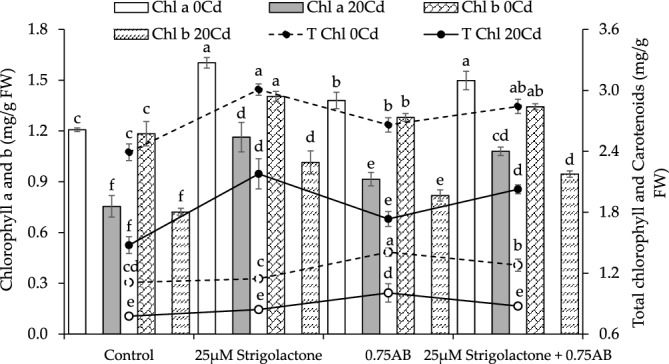
The influence of SLs and biochar on the radish leaves chlorophyll a, b, total and carotenoids grown in no Cd and 20Cd stress. The graph shows the average of 4 replicates with±, where significant differences (*p* ≤ 0.05) are indicated by distinct letters on bars, determined by the Tukey test.

Applying 20 µM SLs, 25 µM SLs + 0.75% AB and 0.75% AB showed ~ 33, ~ 24 and ~ 14% improvement in chlorophyll a content than control at no Cd stress. In 20Cd stress, treatments 25 µM SLs showed a ~ 54%, 25 µM SLs + 0.75% AB caused ~ 43% and 0.75% AB led to ~ 21% improvement in chlorophyll a content over control (Fig. 2).

In no Cd stress, chlorophyll b contents were improved by ~ 19% with 25 µM SLs, ~ 13% with 25 µM SLs + 0.75% AB, and ~ 8% with 0.75% AB over control. Under 20Cd stress, chlorophyll b contents were enhanced by ~ 41% with 25 µM SLs, ~ 31% with 25 µM SLs + 0.75% AB, and ~ 14% with 0.75% AB compared to control (Fig. 2).

The total chlorophyll content improved by ~ 26% in 25 µM SLs, ~ 19% in 25 µM SLs + 0.75% AB and ~ 11% in 0.75% AB over the control at no Cd stress. Under 20Cd stress, 25 µM SLs caused ~ 48%, 25 µM SLs + 0.75% AB resulted in ~ 37% and 0.75% AB showed ~ 18% improvement in total chlorophyll contents over control (Fig. 2).

At no Cd stress, 25 µM SLs led to ~ 3% while 25 µM SLs + 0.75% AB caused ~ 15% improvement in carotenoids over the control. However, 0.75% AB showed ~ 27% enhancement in carotenoids from control under no Cd stress. In 20Cd stress, 25 µM SLs showed an improvement of ~ 8%, 25 µM SLs + 0.75% AB resulted in ~ 13% while 0.75% AB caused ~ 30% enhancement in carotenoids than control (Fig. 2).

### Relative water contents, protein content, and electrolyte leakage

**Figure 3:**
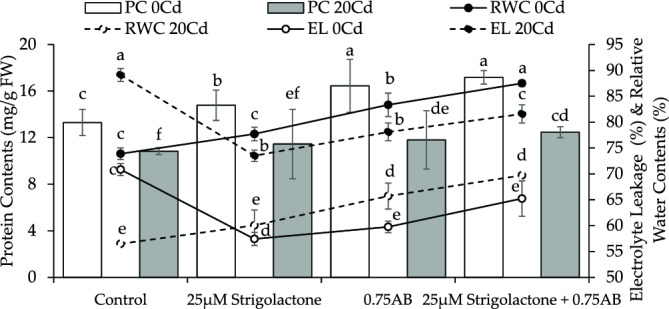
The impact of SLs and biochar on radish protein content, electrolyte leakage, and relative water content grown in no Cd and 20Cd stress. The graph shows the average of 4 replicates with ± , where significant differences (*p* ≤ 0.05) are indicated by distinct letters on bars, determined by the Tukey test.

Treatment 25 µM SLs led to ~ 5% improvement in relative water contents (RWC) from control, while 25 µM SLs + 0.75% AB caused ~ 18% increment in RWC in no Cd stress. The 0.75% AB showed a ~ 13% improvement in RWC over control in no Cd stress. Under 20Cd stress, applying 25 µM SLs caused ~ 6%, 25 µM SLs + 0.75% AB ~ 23% and 0.75% AB led to ~ 16% improvement in RWC than control (Fig. 3A).

The protein content in no Cd stress showed a ~ 11% improvement in 20 µM SLs while ~ 29% where 25 µM SLs + 0.75% AB was applied over control. The addition of 0.75% AB treatment resulted in a ~ 24% improvement in protein content compared to control. In 20Cd stress, protein content showed ~ 6, ~ 15 and ~ 9% improvement in protein content where 25 µM SLs, 25 µM SLs + 0.75% AB and 0.75% AB were applied as treatment respectively than control (Fig. 3B).

Under no Cd stress, 25 µM SLs, 0.75% AB and 25 µM SLs + 0.75% AB led to a ~ 19, ~ 16 and ~ 8% decrease in electrolyte leakage (EL) respectively, compared to control. In 20Cd stress, 25 µM SLs led to a ~ 18%, 25 µM SLs + 0.75% AB caused ~ 8% while 0.75% AB resulted in ~ 12% decrease in EL over control (Fig. 3C).

### Proline content, H_2_O_2_, and MDA

**Figure 4:**
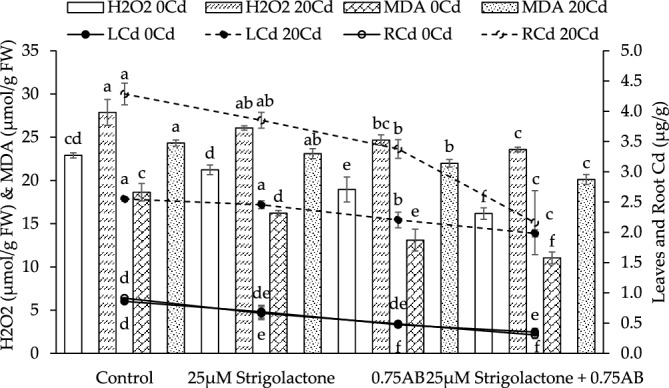
The impact of SLs and biochar on radish H_2_O_2_, malondialdehyde, leaf and root Cd concentration grown in no Cd and 20Cd stress. The graph shows the average of 4 replicates with±, where significant differences (*p* ≤ 0.05) are indicated by distinct letters on bars, determined by the Tukey test.

Results showed that H_2_O_2_ was decreased ~ 7, ~ 29 and ~ 17% when 25 µM SLs, 25 µM SLs + 0.75% AB and 0.75% AB were applied as treatment respectively over control at no Cd stress. In 20Cd stress, H_2_O_2_ was declined ~ 6, ~ 15 and ~ 11% in 25 µM SLs, 25 µM SLs + 0.75% AB and 0.75% AB respectively than control (Fig. 4).

It was noted that 25 µM SLs reduced ~ 13%, 25 µM SLs + 0.75% AB ~ 41% and 0.75% AB ~ 30% MDA over control at no Cd stress. In case of 20Cd stress, 25 µM SLs decreased ~ 5%, 25 µM SLs + 0.75% AB ~ 17% and 0.75% AB ~ 10% MDA compared to control (Fig. 4).

In case of leaves Cd concentration was decreased ~ 20, ~ 59 and ~ 145% when 25 µM SLs, 25 µM SLs + 0.75% AB and 0.75% AB were applied as treatment respectively over control at no Cd stress. In 20Cd stress, leaves Cd concentration was declined ~ 4, ~ 22 and ~ 14% in 25 µM SLs, 25 µM SLs + 0.75% AB and 0.75% AB respectively than control (Fig. 4).

It was noted that 25 µM SLs reduced ~ 27%, 25 µM SLs + 0.75% AB ~ 67% and 0.75% AB ~ 47% roots Cd concentration over control at no Cd stress. In case of 20Cd stress, 25 µM SLs decreased ~ 10%, 25 µM SLs + 0.75% AB ~ 50% and 0.75% AB ~ 21% roots Cd concentration compared to control (Fig. 4).

### Antioxidants activities

**Figure 5:**
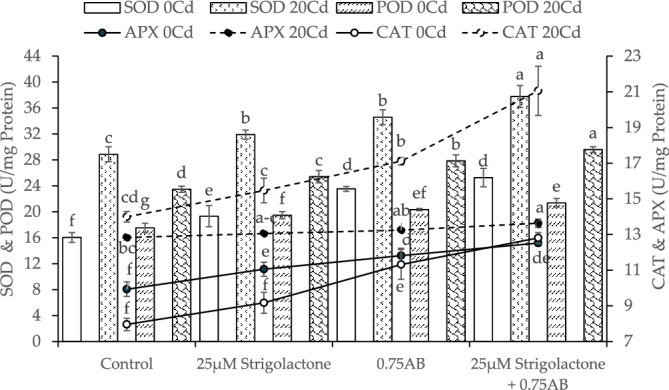
The impact of SLs and biochar on superoxide dismutase (SOD), peroxidase (POD), catalase (CAT), and ascorbate peroxidase (APX) levels in radish grown in no Cd stress and 20Cd stress. The graph shows the average of 4 replicates with±, where significant differences (*p* ≤ 0.05) are indicated by distinct letters on bars, determined by the Tukey test.

When compared to control, the addition of 25 µM SLs led to ~ 20%, 25 µM SLs + 0.75% AB caused ~ 57% while 0.75% AB showed ~ 47% decrease in SOD activity than control. In the 20Cd stress, a decrease of ~ 11, ~ 31 and ~ 20% in SOD activity was observed in 25 µM SLs, 25 µM SLs + 0.75% AB and 0.75% AB compared to control (Fig. 5).

In no Cd stress, treatment 25 µM SLs resulted in ~ 11%, 25 µM SLs + 0.75% AB led to ~ 22% and 0.75% AB caused ~ 16% decrease in POD activity than control under no Cd stress. At 20Cd stress, applying 25 µM SLs showed ~ 8%, 25 µM SLs + 0.75% AB demonstrated ~ 26% while AB exhibited ~ 19% decrease in POD activity, compared to control (Fig. 5).

Results showed a ~ 15, ~ 61 and ~ 42% decline in CAT activity compared to control where 25 µM SLs, 25 µM SLs + 0.75% AB and 0.75% AB were applied respectively. In case of 20Cd stress, 25 µM SLs, 25 µM SLs + 0.75% AB and 50% caused ~ 11, ~ 51 and ~ 22% decrease in CAT activity over control respectively (Fig. 5).

In case of no Cd stress, APX activity was declined up to ~ 38, ~ 88 and ~ 64% in 20 µM SLs, 25 µM SLs + 0.75% AB and 0.75% AB over control respectively. At 20Cd stress, SLs resulted in a ~ 4%, 25 µM SLs + 0.75% AB caused ~ 14% and 0.75% AB showed ~ 7% decrease in APX activity than control (Fig. 5).

### Convex hull and hierarchical cluster analysis

**Figure 6:**
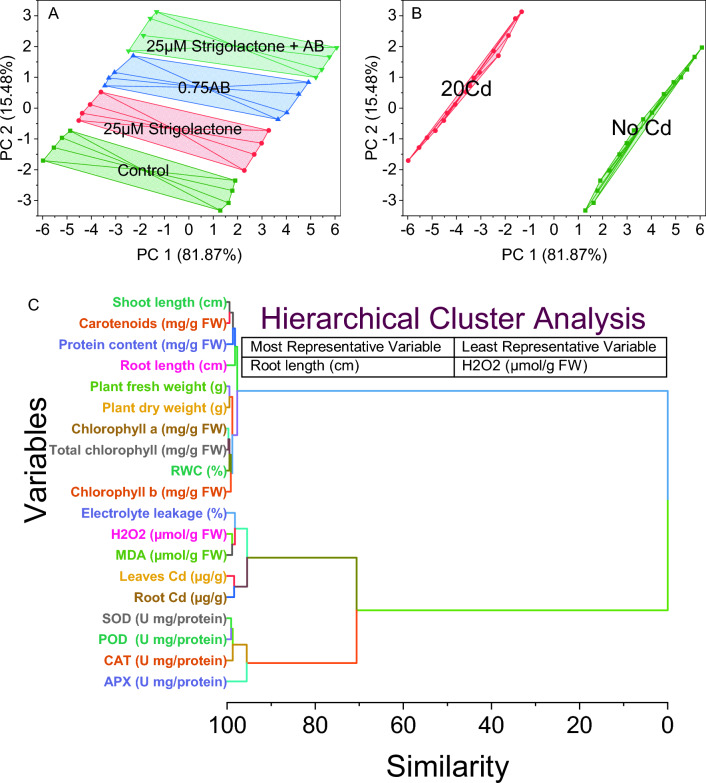
Cluster plot convex hull for treatments (**A**), Cd levels (**B**), and hierarchical cluster plot (**C**) for studied attributes.

Data points are marked with their respective treatment groups: Control, 25 µM SLs, 0.75AB, and 25 µM SLs + AB. The Control group showed a tight cluster characterized by data points with predominantly negative scores on both PC 1 and PC 2. Conversely, the 25 µM SLs treatment group forms a marked cluster, with data points showing predominantly positive scores on both PC 1 and PC 2, indicative of a treatment-specific response. Positioned between the Control and 25 µM SLs groups, the 0.75AB treatment group occupies a distinct section, suggesting a response that deviates from both treatments. Notably, the 25 µM SLs + AB treatment group forms a unique cluster, positioned prominently in the positive region of PC 1 and PC 2, underscoring its distinctive response to the combined treatment (Fig. 6A).

In this analysis, two main clusters are identified, which correspond to different heavy metal (HM) treatments. The first cluster, labeled no Cd, encompasses data points characterized by positive values along both PC 1 and PC 2 axes. These data points exhibit increasing values along both principal components. The convex hull surrounding these points describes the boundary of this cluster, indicating the range of variation within this group. The second cluster, labeled 20Cd, includes data points characterized by negative values along both PC 1 and PC 2 axes. These data points exhibit decreasing values along both principal components (Fig. 6B).

Chlorophyll a and total chlorophyll are tightly associated, evidenced by their similarity score of 0.30045, indicating a close relationship between these two variables. RWC is clustered with a high similarity score of 0.50822, indicating a strong link to physiological parameters concerning water content. Plant fresh weight and plant dry weight exhibit a strong relationship, clustered tightly with a similarity score of 0.51475, representing plant biomass. Shoot length and carotenoids also demonstrate a notable similarity score of 0.52585, indicating shared patterns within the dataset. Chlorophyll b clusters with SOD and POD, suggesting a potential correlation between chlorophyll content and enzymatic antioxidant activities. H_2_O_2_ and MDA form a cluster with a high similarity score of 1.13996, indicating a possible association between these oxidative stress markers. CAT is grouped with root length, indicating a potential relationship between catalase activity and root development. Protein content stands separately with a similarity score of 1.321, suggesting distinct patterns compared to other variables. Leaves Cd and root Cd exhibit a high similarity score of 1.58953, suggesting a close association between cadmium concentrations in leaves and roots. Electrolyte leakage and APX are grouped with a similarity score of 1.76274, indicating at a potential link between electrolyte leakage and ascorbate peroxidase activity. Moreover, clusters with very high similarity scores, including one exceptionally high score (97.71591), denote distinct patterns or relationships within those clusters (Fig. 6C).

### Pearson correlation analysis

**Figure 7:**
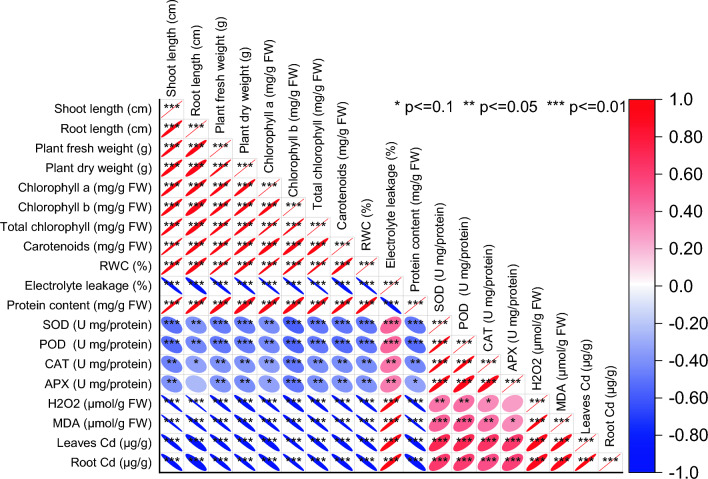
Pearson correlation for studied attributes.

Shoot length exhibited a perfect positive correlation with itself, as expected. Root length showed a very strong positive correlation with shoot length (r = 0.97577), indicating that as shoot length increases, root length tends to increase proportionally. Moreover, plant fresh weight and plant dry weight exhibited very strong positive correlations with both shoot and root lengths, indicating a strong association between plant size and biomass (r = 0.97994–0.99111). Chlorophyll (a. b, and total) and carotenoids all showed very strong positive correlations with each other and with plant biomass measures (r = 0.96005–0.99481). Conversely, electrolyte leakage, a measure of cellular membrane integrity, showed strong negative correlations with plant size, chlorophyll content, and biomass (r = − 0.967 to − 0.98827). Enzymatic antioxidant activities, such as superoxide dismutase (SOD), peroxidase (POD), catalase (CAT), and ascorbate peroxidase (APX), demonstrated negative correlations with most growth and physiological parameters, indicating their involvement in response to stress conditions (r =  − 0.50606 to − 0.38464). Hydrogen peroxide (H_2_O_2_) and malondialdehyde (MDA), markers of oxidative stress, also exhibited strong negative correlations with most parameters, indicating their association with plant health and stress response (r = − 0.98106 to − 0.97233). Additionally, heavy metal accumulation in leaves and roots, represented by leaves Cd and root Cd, displayed strong negative correlations with various growth and physiological parameters, suggesting their detrimental effects on plant growth and health (r = − 0.95299 to − 0.93952) (Fig. 7).

## Discussion

In current study a significant decline in root length, shoot length, plant fresh weight, plant dry weight was chlorophyll contents observed in radish plants which were cultivated in 20 mg Cd/kg soil toxicity. This decline was associated with oxidative stress induced by Cd contamination^37^. Cadmium primarily accumulates in plant roots. High concentrations of Cd in the soil can inhibit root growth directly by damaging root cells and impairing root development^38^. This inhibition can occur due to the disruption of cell division, elongation, and differentiation processes in the root tips. As a result, the overall root length decreases^39^. One of the reasons for reduced shoot length is the impairment of water and nutrient uptake due to damaged roots. Cadmium can interfere with the uptake of essential nutrients such as iron, magnesium, and calcium, which are crucial for plant growth and development^40^. Moreover, Cd can also disrupt photosynthesis by affecting chlorophyll synthesis and photosystem functioning, leading to reduced biomass production and consequently, shorter shoots^41^. Cadmium interferes with various metabolic processes, including photosynthesis, respiration, and protein synthesis, leading to decreased plant productivity and biomass accumulation^42^. Additionally, Cd can induce oxidative stress by generating reactive oxygen species (ROS) and disrupting antioxidant defense mechanisms. This oxidative stress damages cellular structures and biomolecules, further contributing to reduced biomass production and weight^43,44^.

SLs have been implicated in regulating root system architecture, particularly in response to nutrient availability and environmental stresses^13^. By modulating root architecture, SLs can potentially enhance root growth and function even in the presence of Cd toxicity. It can promote the formation of lateral roots and increase root hair density, which may improve nutrient and water uptake efficiency and mitigate the negative impact of Cd on root growth. Additionally, the application of SLs can enhance photosynthesis by stimulating the development of chloroplasts, which in turn results in an increase in chlorophyll content^45^. This hormone is believed to likely stimulate the expression of genes responsible for chlorophyll biosynthesis and maintenance. Steroglactone and AB promote the generation of secondary compounds, such as carotenoids. These compounds are vital antioxidants and play a crucial role in shielding against photodamage^46^. The study found that the combination of biochar and SLs enhanced antioxidant activities under Cd stress, including POD, SOD, CAT, and APX. These enzymes play a crucial role in reducing reactive oxygen species (ROS) levels in plants. CAT decomposes hydrogen peroxide into water and oxygen, while SOD converts superoxide radicals into H2O2 and oxygen. POD and APX both function in the detoxification of H_2_O_2_. These enzymes collectively contribute to the breakdown of ROS, preventing oxidative damage to cellular components such as lipids, proteins, and DNA^47^.

Biochar has a high surface area and contains functional groups that can adsorb and immobilize Cd ions^2,5^. By binding to the surface of biochar, Cd becomes less mobile in the soil and is less likely to be taken up by plant roots. It may compete with plant roots for Cd uptake by adsorbing Cd ions onto its surface. This competitive sorption reduces the concentration of Cd ions available for plant uptake, further decreasing Cd accumulation in plant tissues^48^. Furthermore, acidified biochar can improve soil structure, water retention, and nutrient availability, which indirectly affects plant growth and Cd uptake^49^. Healthier plants grown in well-structured soils may exhibit reduced Cd uptake due to improved physiological conditions. It also serves as a habitat and substrate for soil microorganisms. Microbes associated with biochar can contribute to the biodegradation of organic contaminants and the immobilization of heavy metals through various biological processes. Enhanced microbial activity can contribute to the transformation and sequestration of Cd in the soil, reducing its availability to plants^50–52^.

## Conclusion

In conclusion, the combined application of 0.75% AB and SLs (25 µM) demonstrates promising efficacy in mitigating soil cadmium (Cd) toxicity and enhancing radish cultivation when compared to their individual application and control. The observed reduction in the levels of antioxidants SOD, POD, and CAT under cadmium toxicity (20 mg/kg) with the application of AB and SLs suggests a lower uptake of cadmium in plants. Consequently, the application of AB and SLs in cadmium-contaminated soils hold potential for mitigating cadmium toxicity and achieving higher radish growth.

## Data Availability

All data generated or analysed during this study are included in this published article.

## References

[1] Sana S, Ramzan M, Ejaz S, Danish S, Salmen SH, Ansari MJ (2024). Differential responses of chili varieties grown under cadmium stress. BMC Plant Biol..

[2] Sheikh L, Younis U, Shahzad AS, Hareem M, Noor Elahi N, Danish S (2023). Evaluating the effects of cadmium under saline conditions on leafy vegetables by using acidified biochar. Pakistan J. Bot..

[3] Anwar T, Shehzadi A, Qureshi H, Shah MN, Danish S, Salmen SH (2023). Alleviation of cadmium and drought stress in wheat by improving growth and chlorophyll contents amended with GA3 enriched deashed biochar. Sci. Rep..

[4] Ahmad Rahi A, Younis U, Ahmed N, Arif Ali M, Fahad S, Sultan H (2022). Toxicity of Cadmium and nickel in the context of applied activated carbon biochar for improvement in soil fertility. Saudi J. Biol. Sci..

[5] Ahmed N, Shah AR, Danish S, Fahad S, Ali MA, Zarei T (2021). Immobilization of Cd in soil by biochar and new emerging chemically produced carbon. J. King Saud Univ. Sci..

[6] Vessey JK (2003). Plant growth promoting rhizobacteria as biofertilizers. Plant Soil.

[7] Yaronskaya E, Vershilovskaya I, Poers Y, Alawady AE, Averina N, Grimm B (2006). Cytokinin effects on tetrapyrrole biosynthesis and photosynthetic activity in barley seedlings. Planta.

[8] Nazli F, Mustafa A, Ahmad M, Hussain A, Jamil M, Wang X (2020). A review on practical application and potentials of phytohormone-producing plant growth-promoting rhizobacteria for inducing heavy metal tolerance in crops. Sustainability.

[9] Li C, Lu X, Liu Y, Xu J, Yu W (2023). Strigolactone alleviates the adverse effects of salt stress on seed germination in cucumber by enhancing antioxidant capacity. Antioxidants.

[10] Gomez-Roldan V, Fermas S, Brewer PB, Puech-Pagès V, Dun EA, Pillot JP (2008). Strigolactone inhibition of shoot branching. Nature.

[11] Soliman S, Wang Y, Han Z, Pervaiz T, El-kereamy A (2022). SLs in plants and their interaction with the ecological microbiome in response to abiotic stress. Plants.

[12] Wani KI, Naeem M, Khan MMA, Aftab T (2023). Insights into strigolactone (GR24) mediated regulation of cadmium-induced changes and ROS metabolism in *Artemisia annua*. J. Hazard Mater..

[13] Raja V, Qadir SU, Kumar N, Alsahli AA, Rinklebe J, Ahmad P (2023). Melatonin and strigolactone mitigate chromium toxicity through modulation of ascorbate-glutathione pathway and gene expression in tomato. Plant Physiol. Biochem..

[14] Younis U, Danish S, Datta R, Alahmadi TA, Ansari MJ (2024). Sustainable remediation of chromium-contaminated soils: Boosting radish growth with deashed biochar and strigolactone. BMC Plant Biol..

[15] Huang S, Huang P, Hareem M, Tahzeeb-ul-Hassan M, Younis U, Dawar K (2024). Evaluating the hidden potential of deashed biochar in mitigating salinity stress for cultivation of fenugreek. Sci. Rep..

[16] Shahzad AS, Younis U, Naz N, Danish S, Syed A, Elgorban AM (2023). Acidified biochar improves lead tolerance and enhances morphological and biochemical attributes of mint in saline soil. Sci. Rep..

[17] Hareem M, Danish S, Pervez M, Irshad U, Fahad S, Dawar K (2024). Optimizing chili production in drought stress: Combining Zn-quantum dot biochar and proline for improved growth and yield. Sci. Rep..

[18] Ramzan M, Jamshaid T, Ali L, Dawar K, Saba R, Jamshaid U (2023). Modulation of sunflower growth via regulation of antioxidants, oil content and gas exchange by arbuscular mycorrhizal fungi and quantum dot biochar under chromium stress. BMC Plant Biol..

[19] Anwar S, Shah AA, Hussain NA, Ramzan M, Khan WU, Kousar S (2023). Interactive potential of Bacillus megaterium A12 and biochar in chromium stress mitigation in Spinacia
* oleraceae*: Methylglyoxal detoxification and activation of antioxidant enzymes. Pakistan J. Bot..

[20] Tanwar R, Panghal A, Chaudhary G, Kumari A, Chhikara N (2023). Nutritional, phytochemical and functional potential of sorghum: A review. Food Chem. Adv..

[21] Mukherjee S, Chatterjee N, Sircar A, Maikap S, Singh A, Acharyya S (2023). A comparative analysis of heavy metal effects on medicinal plants. Appl. Biochem. Biotechnol..

[22] Máthé-Gáspár G, Anton A (2002). Heavy metal uptake by two radish varieties. Acta Biol Szeged..

[23] Weatherley P (1950). Studies in the water relations of the cotton plant: I: The field measurement of water deficits in leaves. New Phytol..

[24] Arnon DI (1949). Copper enzymes in isolated chloroplasts polyphenoloxidase in Beta vulgaris. Plant Physiol..

[25] Kirk JTO, Allen RL (1965). Dependence of chloroplast pigment synthesis on protein synthesis: Effect of actidione. Biochem. Biophys. Res. Commun..

[26] Dhindsa RS, Plumb-Dhindsa PL, Reid DM (1982). Leaf senescence and lipid peroxidation: Effects of some phytohormones, and scavengers of free radicals and singlet oxygen. Physiol. Plant..

[27] Hori M, Kondo H, Ariyoshi N, Yamada H, Hiratsuka A, Watabe T (1997). Changes in the hepatic glutathione peroxidase redox system produced by coplanar polychlorinated biphenyls in Ah-responsive and-less-responsive strains of mice: Mechanism and implications for toxicity. Environ. Toxicol. Pharmacol..

[28] Aebi H. Catalase in vitro. 1984. p. 121–6. 10.1016/s0076-6879(84)05016-3 6727660

[29] Nakano Y, Asada K (1981). Hydrogen peroxide is scavenged by ascorbate-specific peroxidase in spinach chloroplasts. Plant Cell Physiol..

[30] Jiang M, Zhang J (2001). Effect of abscisic acid on active oxygen species, antioxidative defence system and oxidative damage in leaves of maize seedlings. Plant Cell Physiol..

[31] Anderson ME (1985). Determination of glutathione and glutathione disulfide in biological samples. Methods Enzymol..

[32] Hodges DM, Andrews CJ, Johnson DA, Hamilton RI (1996). Antioxidant compound responses to chilling stress in differentially sensitive inbred maize lines. Physiol. Plant..

[33] Lutts S, Kinet JM, Bouharmont J (1996). NaCl-induced senescence in leaves of rice (*Oryza sativa* L.) cultivars differing in salinity resistance. Ann. Bot..

[34] Bates LS, Waldren RP, Teare ID (1973). Rapid determination of free proline for water-stress studies. Plant Soil..

[35] Steel RG, Torrie JH, Dickey DA (1997). Principles and Procedures of Statistics: A Biometrical Approach.

[36] OriginLab Corporation. OriginPro. Northampton, MA, USA.: OriginLab; 2021.

[37] Raza SH, Shafiq F, Anwar S (2022). The influence of salicylic acid foliar spray on the growth, biochemical traits, and Cd-uptake in radish (*Raphanus Sativus* L.). Pakistan J Bot..

[38] Kirkham MB (2006). Cadmium in plants on polluted soils: Effects of soil factors, hyperaccumulation, and amendments. Geoderma..

[39] Gratão PL, Polle A, Lea PJ, Azevedo RA (2005). Making the life of heavy metal-stressed plants a little easier. Funct. plant Biol..

[40] Gill SS, Khan NA, Anjum NA, Tuteja N (2011). Amelioration of cadmium stress in crop plants by nutrients management: Morphological, physiological and biochemical aspects. Plant Stress.

[41] Bashir H, Qureshi MI, Ibrahim MM, Iqbal M (2015). Chloroplast and photosystems: Impact of cadmium and iron deficiency. Photosynthetica.

[42] Chen X-X, Xu Y-M, Lau ATY (2022). Metabolic effects of long-term cadmium exposure: an overview. Environ. Sci. Pollut. Res..

[43] Garcia JS, Gratão PL, Azevedo RA, Arruda MAZ (2006). Metal contamination effects on sunflower (*Helianthus annuus* L.) growth and protein expression in leaves during development. J. Agric. Food Chem..

[44] Cuypers A, Plusquin M, Remans T, Jozefczak M, Keunen E, Gielen H (2010). Cadmium stress: An oxidative challenge. BioMetals.

[45] Wani KI, Zehra A, Choudhary S, Naeem M, Khan MMA, Khan R (2023). Exogenous strigolactone (GR24) positively regulates growth, photosynthesis, and improves glandular trichome attributes for enhanced artemisinin production in Artemisia annua. J. Plant Growth Regul..

[46] Lingwan M, Pradhan AA, Kushwaha AK, Dar MA, Bhagavatula L, Datta S (2023). Photoprotective role of plant secondary metabolites: Biosynthesis, photoregulation, and prospects of metabolic engineering for enhanced protection under excessive light. Environ. Exp. Bot..

[47] Pandey N, Singh GK (2012). Studies on antioxidative enzymes induced by cadmium in pea plants (*Pisum sativum*). J. Environ. Biol..

[48] Trakal L, Veselská V, Šafařík I, Vítková M, Číhalová S, Komárek M (2016). Lead and cadmium sorption mechanisms on magnetically modified biochars. Bioresour. Technol..

[49] Suppadit T, Kitikoon V, Phubphol A, Neumnoi P (2012). Effect of quail litter biochar on productivity of four new physic nut varieties planted in cadmium-contaminated soil. Chil. J. Agric. Res..

[50] Zafar-ul-Hye M, Tahzeeb-ul-Hassan M, Wahid A, Danish S, Khan MJ, Fahad S (2021). Compost mixed fruits and vegetable waste biochar with ACC deaminase rhizobacteria can minimize lead stress in mint plants. Sci. Rep..

[51] Zafar-Ul-Hye M, Danish S, Abbas M, Ahmad M, Munir TM (2019). ACC deaminase producing PGPR Bacillus amyloliquefaciens and agrobacterium fabrum along with biochar improve wheat productivity under drought stress. Agronomy.

[52] Zafar-ul-Hye M, Tahzeeb-ul-Hassan M, Abid M, Fahad S, Brtnicky M, Dokulilova T (2020). Potential role of compost mixed biochar with rhizobacteria in mitigating lead toxicity in spinach. Sci. Rep..

